# Therapeutic Efficacy and Safety of Traditional Chinese Medicine Classic Herbal Formula *Longdanxiegan* Decoction for Hypertension: A Systematic Review and Meta-Analysis

**DOI:** 10.3389/fphar.2018.00466

**Published:** 2018-05-08

**Authors:** Xing-jiang Xiong, Xiao-chen Yang, Wei Liu, Lian Duan, Peng-qian Wang, Hu You, Xiao-ke Li, Shihan Wang

**Affiliations:** ^1^Department of Cardiology, Guang'anmen Hospital, China Academy of Chinese Medical Sciences, Beijing, China; ^2^Yale Cardiovascular Research Center, Yale School of Medicine, Yale University, New Haven, CT, United States; ^3^Department of Cardiology, Traditional Chinese Medicine Hospital of Beijing, Capital Medical University, Beijing, China; ^4^Department of Pharmacology, Institute of Chinese Materia Medica, China Academy of Chinese Medical Sciences, Beijing, China; ^5^Department of Chinese Medicine, Nanjing Benq Hospital, Nanjing Medical University, Nanjing, China; ^6^Department of Treatise on Febrile Diseases, College of Basic Medicine, Nanjing University of Chinese Medicine, Nanjing, China; ^7^Bio-organic and Natural Products Laboratory, McLean Hospital, Harvard Medical School, Belmont, MA, United States

**Keywords:** hypertension, blood pressure, traditional Chinese medicine, classic herbal formula, *Longdanxiegan* decoction, systematic review

## Abstract

**Background:** The traditional Chinese medicine classic herbal formula *Longdanxiegan* decoction (LDXGD) is widely used for hypertensive patients in China.

**Objectives:** This systematic review and meta-analysis aimed to evaluate the efficacy and safety of LDXGD for hypertension.

**Methods:** PubMed, EMBASE, Cochrane Central Register of Controlled Trials, Chinese Biomedical Literature Database, Chinese Scientific Journal Database, Chinese National Knowledge Infrastructure, and Wanfang Database were searched up to February 7, 2017 for randomized control trials in treating hypertension.

**Results:** Nine trials were identified. Compared with antihypertensive drugs, Longdanxiegan decoction plus antihypertensive drugs (LPAD) significantly improved systolic blood pressure (BP) (*n* = 138; MD = −4.82 mmHg; 95% CI: −7.89 to −1.76; *P* = 0.002), diastolic BP (*n* = 138; MD = −2.42 mmHg; 95% CI: −3.22 to −1.62; *P* < 0.00001), categorical BP (*n* = 509; RR: 1.26; 95% CI: 1.17 to 1.36; *P* < 0.00001), hypertension related symptoms (*n* = 509; RR: 1.31; 95% CI: 1.15 to 1.49; *P* < 0.0001), and heart rate (*n* = 138; MD = −2.40 bpm; 95% CI: −4.23 to −0.56; *P* = 0.01). Beneficial effects but no statistically significant reduction in total cholesterol (*n* = 138; MD = −0.11 mmol/l; 95% CI: −0.65 to 0.44; *P* = 0.71), or triglyceride (*n* = 138; MD = −0.20 mmol/l; 95% CI: −0.46 to 0.07; *P* = 0.14) was observed in LPAD. Compared with antihypertensive drugs, LDXGD used alone significantly improved systolic BP, diastolic BP, and hypertension related symptoms. But there was no difference between LDXGD and antihypertensive drugs on categorical BP (*n* = 120; RR: 1.09; 95% CI: 0.96 to 1.23; *P* = 0.18). The safety of LDXGD were still unclear.

**Conclusions:** Due to poor methodological quality of the included trials, as well as potential reporting bias, our review found no conclusive evidence for the effectiveness of LDXGD in treating hypertension. The potential beneficial effects and safety of LDXGD should be assessed in future properly designed trials.

## Introduction

Cardiovascular diseases continue to represent the major cause of death worldwide. Hypertension is a major contributor to cardiovascular diseases, cerebrovascular disease, and renal failure (James et al., 2014). A strong association between hypertension and cardiovascular diseases has been established by numerous epidemiological studies. Over the past 2 decades, abundant evidence from randomized controlled trials (RCTs) identified that the current awareness, curative, and control rates of hypertension have been improved; however, they remain far from ideal (Unger, 2014). Thus, some hypertensive patients have turned to traditional Chinese medicine (TCM) for seeking new antihypertensive modalities with fewer adverse effects in East Asia (Xiong et al., 2013a).

TCM classic herbal formula is defined as a formula that has been recorded in ancient TCM classic medical books with fixed herbal drug composition, definite curative effect, and fewer adverse effects for certain diseases (Xiong et al., 2017a). Recently, more robust evidence from meta-analyses and systematic reviews have showed improvements in blood pressure (BP) and hypertension related symptoms by TCM classic herbal formulas, including *Banxia baizhu tianma* decoction (Xiong et al., 2012), *Tianma gouteng* yin (Wang et al., 2013), *Zhen gan xi feng* decoction (Xiong et al., 2013b), *Jian ling* decoction (Xiong et al., 2015a), *Liu wei di huang* wan (Wang et al., 2012), *Shenqi* pill (Xiong et al., 2015b), *Zhen wu* decoction (Xiong et al., 2015c), and *Xuefu zhuyu* decoction (Wang et al., 2015). *Longdanxiegan* decoction (LDXGD) is one of the most utilized TCM classic herbal formula invented by *Ang Wang* in *Yi Fang Ji Jie* (Variorum of Medical Recipes) in the *Qing* Dynasty. According to TCM theory, liver-*yang* hyperactivity syndrome and liver damp-heat syndrome are 2 most common patterns of hypertension, which often appear simultaneously (Wang and Xiong, 2012). Interestingly, LDXGD possesses the efficacy of calming the liver-*yang* and clearing away liver damp-heat, which could obviously improve the liver-*yang* hyperactivity and liver damp-heat syndromes in TCM theory. Such pharmacological effects of LDXGD amount to inhibition of sympathetic activity from the perspective of modern science. LDXGD is composed by the following 10 Chinese herbal medicines, including Chinese Gentian Root (*Longdancao*, Radix Gentianae Longdancao), Gardenia (*Zhizi*, Fructus Gardeniae Jasminoidis), Baical Skullcap Root (*Huangqin*, Radix Scutellariae Baicalensis), Thorowax Root (*Chaihu*, Radix Bupleuri), Rehmannia (*Dihuang*, Radix Rehmanniae Glutinosae), Plantain Seed (*Cheqianzi*, Semen Plantaginis), Alisma (*Zexie*, Rhizoma Alismatis), Akebia Stem (*Mutong*, Caulis Akebiae), Chinese Angelica Root (*Danggui*, Radix Angelicae Sinensis), and Liquoric Root (*Gancao*, Radix Glycyrrhizae). The main chemical components of LDXGD included gentiopicrin, gardenoside, baicalin, rehmannioside, plantaginin, alisol A monoacetate, angelica polysaccharide, etc (Wang et al., 2007). Currently, it has been widely used to treat numerous diseases including acute cholecystitis, non-alcoholic fatty liver disease (Yu and Zhang, 2017), hyperthyroidism (Lei and Wei, 2017), acute posterior ganglionitis (Wang and Wang, 2016), acute epididymitis (Pan et al., 2016), drug hepatitis (Zhu, 2016), hypertension (Lv, 2007), etc.

In recent years, LDXGD has been widely prescribed in treating hypertension by TCM physicians in China. The indications of LDXGD for hypertensive patients included vertigo, headache, facial flushing with perspiration, conjunctival congestion, bitter taste in the mouth, thirst, irritability and restlessness, red tongue with yellow greasy fur, wiry-rapid-powerful pulse or powerful cunkou pulse alone, or wiry and long pulse even well-beyond the cunkou pulse. This prescription can be used as long as it meets the above indications regardless of the level and risk stratification of hypertension by western medicine. Numerous clinical studies ranged from case reports, case series to RCTs also suggested that LDXGD might be effective in lowering BP and relieving hypertension related symptoms (Yang, 2013; Shi, 2014; Wang, 2014). However, the clinical benefit of LDXGD for hypertension still remains uncertain due to limited sample size, inconsistent trial design, and unclear methodological quality of the published clinical trials. Therefore, we conducted this systematic review and meta-analysis for the following purposes: (a) to examine the efficacy of LDXGD vs. placebo, no intervention, or antihypertensive drugs; (b) to examine the effectiveness of LDXGD plus antihypertensive drugs (LPAD) vs. antihypertensive drugs used alone; and (c) to examine the safety of LDXGD.

## Methods

This systematic review and meta-analysis was performed in accordance with the Preferred Reporting Items for Systematic Reviews and Meta-Analyses (PRISMA) guidelines (Liberati et al., 2009).

### Search strategies for identification of studies

We searched PubMed, EMBASE, Cochrane Central Register of Controlled Trials, Chinese Biomedical Literature Database, Chinese Scientific Journal Database, Chinese National Knowledge Infrastructure, and Wanfang Database from their inceptions to February 7, 2017 to identify RCTs that examined the efficacy of LDXGD on hypertension. We used the following keywords for databases searching: (“hypertension” OR “essential hypertension” OR “primary hypertension” OR “high blood pressure” OR “blood pressure” OR “gao xue ya”) AND (“Longdanxiegan decoction” OR “Longdanxiegan tang” OR “Longdanxiegantang”) AND (“clinical trial” OR “randomized controlled trial” OR “randomized controlled trial” OR “lin chuang yan jiu”). Reference of important articles were searched manually for possible relevant studies. No restriction on publication status or language was preestablished.

### Inclusion criteria

Only RCTs that met the following criteria could be enrolled in this review: (a) the trial evaluated the efficacy of LDXGD for hypertension; (b) patients were diagnosed by essential hypertension based on the diagnostic criteria of Chinese Guidelines for the Management of Hypertension, Guidelines of Clinical Research of New Drugs of Traditional Chinese Medicine, WHO-ISH Guidelines for the Management of Hypertension, The seventh report of the Joint National Committee on Prevention, Detection, Evaluation and Treatment of high blood pressure (JNC 7), and other international standards; (c) LDXGD was the only active intervention in the treatment group compared with a control group, including placebo, no intervention, and antihypertensive drugs; (d) RCTs compared LPAD with antihypertensive drugs used alone; (e) in studies comparing LPAD with antihypertensive drugs, the type, specifications, and dosage of antihypertensive drugs used in the treatment groups were the same as used in the control groups; (f) the primary outcome measure was defined as BP, including systolic blood pressure (SBP), diastolic blood pressure (DBP), and categorical BP; and (f) the secondary outcome measures were defined as mortality, progression to severe complications, symptoms, heart rate (HR), total cholesterol (TC), and triglycerides (TG). Severe complications were defined as coronary heart disease, acute myocardial infarction, heart failure, acute cerebrovascular disease, hypertensive renal damage, and retinopathy due to high BP level. Hypertension related symptoms recommended by China Food and Drug Administration included vertigo, headache, etc. No limitation on gender, age, or ethnic origin was provided. As the rapidly antihypertensive effect of LDXGD was emphasized in some studies, no restrictions on treatment duration was preestablished.

### Exclusion criteria

If the following conditions were identified, the study should be excluded: (a) studies were were not randomized or did not adopt a control arm; (b) patients were diagnosed as secondary hypertension, including renal hypertension, cushing syndrome, primary aldosteronism, pheochromocytoma, and abdominal aortic banding; (c) other interventions including *qigong*, yoga, *Tai Chi, Baduanjin*, acupuncture, cupping, moxibustion, massage, and blood-letting therapy were utilized in either treatment or control group; and (d) no data on BP was reported.

### Data extraction

The data was independently extracted by two reviewers (WL and LD) according to the predesigned inclusion and exclusion criteria. If disagreement was identified, it was resolved by discussion with the third party (XKL and SHW). The following data was extracted from the original research: first author's name, title, publication year, sample size, age, sex distribution, diagnostic criteria, study design, methods used to generate the randomization, level of blinding, explanation for withdrawal or dropout, intervention methods, composition of LDXGD or modified LDXGD, treatment duration, outcome measures, and adverse effects. We also contacted the original authors for missing information about the studies by emails, Telephone, or fax.

### Risk of bias assessment

Two reviewers (PQW and HY) independently evaluated the risk of bias of the included individual study according to the Cochrane Handbook for Systematic Reviews of Interventions (Higgins and Green, 2011). If missing data about the study design was identified, the corresponding authors were contacted through telephone, email, or fax contained in either original research or published protocol. The following 7 aspects were evaluated: (a) random sequence generation (selection bias); (b) allocation concealment (selection bias); (c) blinding of participants and personnel (performance bias); (d) blinding of outcome assessment (detection bias); (e) incomplete outcome data (attrition bias); (f) selective reporting (reporting bias); and (g) other sources of bias (from Chapter 8: assessing risk of bias in included studies). Judgments of each item were categorized as “low risk of bias,” “high risk of bias,” or “unclear risk of bias” according to the above criteria. Any discrepancy was resolved by the third party (XKL and SHW).

### Data synthesis and analysis

Review Manager software (RevMan, Version 5.3, Copenhagen: The Nordic Cochrane Centre, Copenhagen, Denmark, The Cochrane Collaboration, 2014) was utilized for data synthesis and analysis. For continuous outcomes, they were presented as mean difference (MD) between intervention and control groups with 95% confidence interval. And for dichotomous data, they were presented as relative risk (RR) with 95% confidence interval. In this meta-analysis, SBP, DBP, HR, TC, and TG were presented as MD, while categorical BP, symptoms, and adverse effects as RR. The random-effects model rather than the fixed-effects model was used because the heterogeneity between multi-studies has to be taken into account. *P* < 0.05 was considered to be of statistically significance.

## Results

### Study identification

**Figure 1:**
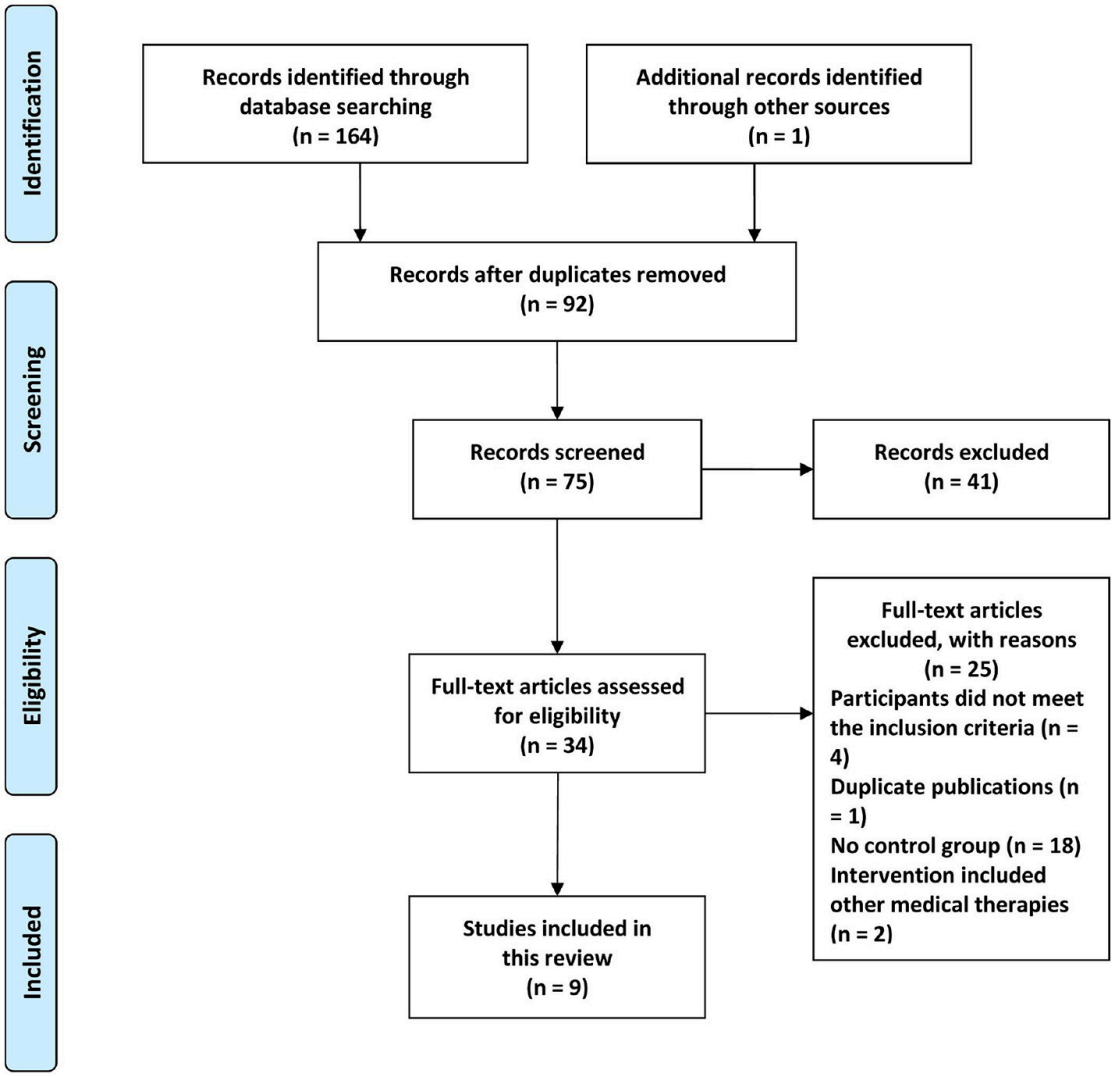
Flow diagram of study selection and identification.

According to our search strategy in 7 electronic databases, 165 potential relevant publications were searched, of which 34 were retrieved for further assessment, and 25 were excluded for the reasons listed in Figure 1. Finally, 9 eligible studies met the inclusion criteria and were included (Yu and Ma, 2004; He and Wang, 2010; Ye, 2013; Cao et al., 2014; Chen et al., 2014; Li, 2014, 2015; Xu, 2014; Ma, 2015).

### Study characteristics

The detailed information of basic characteristics was summarized in Table 1. All of the 9 studies included were single-centered, randomized, parallel, controlled clinical trials conducted in China and published in Chinese language. No cross-over design was applied. A total of 863 patients with hypertension were enrolled in the review, with the sample size ranged from 42 to 241. All trials evaluated the efficacy of LDXGD used alone or LPAD compared with antihypertensive drugs in hypertension treatment. Patients in the treatment groups were treated by LDXGD alone or LPAD. The components of LDXGD or modified LDXGD were depicted in Table 2. Patients in the control groups were treated by antihypertensive drugs alone. Treatment duration ranged from 3 days to 12 weeks. BP data was reported in all trials. The primary outcome measure on SBP and DBP were reported in 3 trials (Ye, 2013; Li, 2014, 2015), while categorical BP was reported in 6 trials (Yu and Ma, 2004; He and Wang, 2010; Cao et al., 2014; Chen et al., 2014; Xu, 2014; Ma, 2015). The secondary outcome measures were also reported. Among them, the symptoms were reported in 6 trials (Yu and Ma, 2004; He and Wang, 2010; Cao et al., 2014; Chen et al., 2014; Xu, 2014; Ma, 2015), while HR, TC, and TG were reported in 2 trials (Ye, 2013; Li, 2015).

**Table 1:** Basic characteristics of the included trials.

**References**	**Sample size (randomized/analyzed) M/F**	**Age(yrs)**	**Diagnosis standard**	**Intervention**	**Control**	**Treatment duration**	**Baseline difference**	**Adverse effects reporting**	**Outcome measures**
Ye, 2013	42/42 T: 21 C: 21 F/M: NR	43–77 (T/C: NR)	CGMH-2010	LDXGD (1 dose/d)+C	hydrochlorothiazide (50 mg, bid)	12 weeks	NSD	N	SBP, DBP, HR, TC, and TG
Li, 2015	96/96 T: 48 C: 48 F/M: NR	53.23 ± 2.84 (T/C: NR)	CGMH-2010	LDXGD (1 dose/d)+C	hydrochlorothiazide (50 mg, bid)	12 weeks	NSD	N	SBP, DBP, HR, TC, and TG
Chen et al., 2014	100/100 T: 50 C: 50 F/M: NR	35–80 (T/C: NR)	NR	modified LDXGD (300-400 ml/d)+C	nifedipine controlled release tablet (20 mg, qd) or nifedipine tablet (10 mg, bid)	6 weeks	NSD	N	BP and symptoms
Cao et al., 2014	60/60 T: 15/15 C: 16/14	T: 53.27 ± 9.20 C: 51.00 ± 11.29	CGMH-2010	modified LDXGD (100 ml, tid)+C	sodium nitroprusside	3 days	NSD	N	BP and symptoms
Xu, 2014	108/108 T: 40/14 C: 38/16	T: 32–85 C: 33-84	WHO -ISH GMH-1999	LDXGD (1 dose/d)+C	amlodipine tablet, candesartan, metoprolol, or extended release nifedipine tablet	4 weeks	NSD	N	BP and symptoms
Ma, 2015	241/241 T: 72/49 C: 68/52	T: 33–75 C: 32–77	NR	modified LDXGD (1 dose/d)+C	sodium nitroprusside	3 days	NSD	N	BP and symptoms
Li, 2014	96/96 T: 48 C: 48 F/M: NR	50.36 ± 2.14 (T/C: NR)	CGMH-2010	LDXGD (1 dose/d)	hydrochlorothiazide (50 mg, bid)	12 weeks	NSD	Y	SBP and DBP
Yu and Ma, 2004	60/60 T: 18/12 C: 17/13	T: 63 C: 66	WHO -ISH GMH-1999	modified LDXGD (200 ml, tid)/d	captopril (25 mg, tid)	3 weeks	NSD	Y	BP and symptoms
He and Wang, 2010	60/60 T: 17/13 C: 15/15	T: 52 C: 51	GCRNDTCM	LDXGD (150 ml, qd)/d	extended release nifedipine tablet (20-40 mg, qd)	4 weeks	NSD	Y	BP and symptoms

*BP, blood pressure; C, control group; CGMH, Chinese Guidelines for the Management of Hypertension; DBP, diastolic blood pressure; F, female; GCRNDTCM, Guidelines of Clinical Research of New Drugs of Traditional Chinese Medicine; HR, heart rate; LDXGD, longdanxiegan decoction; M, male; N, no; NR, not reported; NSD, no significant difference; SBP, systolic blood pressure; T, treatment group; TC, total cholesterol; TG, triglycerides; WHO-ISH GMH, WHO-ISH guidelines for the management of hypertension; Y, yes*.

**Table 2:** Components of Chinese herbal formulas used in the included trials.

**References**	**Formula**	**Components**
Ye, 2013	LDXGD	Chinese Gentian Root (Longdancao, Radix Gentianae Longdancao) 15 g, Gardenia (Zhizi, Fructus Gardeniae Jasminoidis) 15 g, Baical Skullcap Root (Huangqin, Radix Scutellariae Baicalensis) 10 g, Plantain Seed (Cheqianzi, Semen Plantaginis) 10 g, Akebia Stem (Mutong, Caulis Akebiae) 10 g, Alisma (Zexie, Rhizoma Alismatis) 10 g, Rehmannia (Dihuang, Radix Rehmanniae Glutinosae) 10 g, Chinese Angelica Root (Danggui, Radix Angelicae Sinensis) 5 g, Thorowax Root (Chaihu, Radix Bupleuri) 10 g, and Liquoric Root (Gancao, Radix Glycyrrhizae) 5 g.
Li, 2015	LDXGD	Chinese Gentian Root (Longdancao, Radix Gentianae Longdancao) 15 g, Gardenia (Zhizi, Fructus Gardeniae Jasminoidis) 15 g, Baical Skullcap Root (Huangqin, Radix Scutellariae Baicalensis) 10 g, Plantain Seed (Cheqianzi, Semen Plantaginis) 10 g, Akebia Stem (Mutong, Caulis Akebiae) 10 g, Alisma (Zexie, Rhizoma Alismatis) 10 g, Rehmannia (Dihuang, Radix Rehmanniae Glutinosae) 10 g, Chinese Angelica Root (Danggui, Radix Angelicae Sinensis) 5 g, Thorowax Root (Chaihu, Radix Bupleuri) 10 g, and Liquoric Root (Gancao, Radix Glycyrrhizae) 5 g.
Chen et al., 2014	modified LDXGD	Chinese Gentian Root (Longdancao, Radix Gentianae Longdancao), Gardenia (Zhizi, Fructus Gardeniae Jasminoidis), Baical Skullcap Root (Huangqin, Radix Scutellariae Baicalensis), Thorowax Root (Chaihu, Radix Bupleuri), Rehmannia (Dihuang, Radix Rehmanniae Glutinosae), Plantain Seed (Cheqianzi, Semen Plantaginis), Alisma (Zexie, Rhizoma Alismatis), Akebia Stem (Mutong, Caulis Akebiae), Liquoric Root (Gancao, Radix Glycyrrhizae), Chinese Angelica Root (Danggui, Radix Angelicae Sinensis), Gastrodia (Tianma, Gastrodiae Rhizoma), Gambir Vine Stems and Thorns (Gouteng, Ramulus Uncariae Cum Uncis), Abalone Shell (Shijueming, Haliotidis Concha), Eucommia Bark (Duzhong, Cortex Eucommiae Ulmoidis), Achyranthes Root (Niuxi, Achyranthis Bidentatae Radix), Chinese Taxillus Twig (Sangjisheng, Herba Taxilli), Poria (Fuling, Scierotium Poriae Cocos), and Flowey Knotweed Stem (Yejiaoteng, Polygoni Multiflori Caulis). If constipation was identifed, Magnolia Bark (Houpu, Cortex Magnoliae Officinalis), Immature Bitter Orange (Zhishi, Fructus Citri Seu Ponciri Immaturus), Rhubarb Root and Rhizome (Dahuang, Radix Et Rhizoma Rhei), and Sodium Sulfate Powder (Mangxiao, Natrii Sulfas Exsiccatus) were added. If severe vertigo was identified, Fossilized Mammal Bones (Longgu, Os Draconis) and Oyster Shell (Muli, Concha Ostreae) were added. If hypertnesion or unstable blood pressure was identifed, Antelope Horn (Lingyangjiao, Cornu Saigae Tataricae) was added.
Cao et al., 2014	modified LDXGD	Chinese Gentian Root (Longdancao, Radix Gentianae Longdancao) 30 g, Gardenia (Zhizi, Fructus Gardeniae Jasminoidis) 15 g, Baical Skullcap Root (Huangqin, Radix Scutellariae Baicalensis) 15 g, Ricepaperplant Pith (Tongcao, Medulla Tetrapanacis) 15 g, Alisma (Zexie, Rhizoma Alismatis) 15 g, Plantain Herb (Cheqiancao, Herba Plantaginis) 15 g, Thorowax Root (Chaihu, Radix Bupleuri) 30 g, Liquoric Root (Gancao, Radix Glycyrrhizae) 15 g, Chinese Angelica Root (Danggui, Radix Angelicae Sinensis) 15 g, Rehmannia (Dihuang, Radix Rehmanniae Glutinosae) 30 g, Achyranthes Root (Niuxi, Achyranthis Bidentatae Radix) 30 g, Earthworm (Dilong, Lumbricus) 15 g, Prunella (Xiakucao, Spica Prunellae Vulgaris) 15 g, and Seaweed (Haizao, Sargassum) 15 g.
Xu, 2014	LDXGD	Chinese Gentian Root (Longdancao, Radix Gentianae Longdancao) 15 g, Baical Skullcap Root (Huangqin, Radix Scutellariae Baicalensis) 19 g, Gardenia (Zhizi, Fructus Gardeniae Jasminoidis) 10 g, Alisma (Zexie, Rhizoma Alismatis) 20 g, Akebia Stem (Mutong, Caulis Akebiae) 10 g, Plantain Seed (Cheqianzi, Semen Plantaginis) 10 g, Chinese Angelica Root (Danggui, Radix Angelicae Sinensis) 12 g, Rehmannia (Dihuang, Radix Rehmanniae Glutinosae) 20 g, Thorowax Root (Chaihu, Radix Bupleuri) 10 g, and Liquoric Root (Gancao, Radix Glycyrrhizae) 10 g.
Ma, 2015	modified LDXGD	Baical Skullcap Root (Huangqin, Radix Scutellariae Baicalensis) 15 g, Gardenia (Zhizi, Fructus Gardeniae Jasminoidis) 15 g, Thorowax Root (Chaihu, Radix Bupleuri) 15 g, Alisma (Zexie, Rhizoma Alismatis) 15 g, Plantain Herb (Cheqiancao, Herba Plantaginis) 15 g, Chinese Angelica Root (Danggui, Radix Angelicae Sinensis) 15 g, Prunella (Xiakucao, Spica Prunellae Vulgaris) 15 g, Ricepaperplant Pith (Tongcao, Medulla Tetrapanacis) 15 g, Seaweed (Haizao, Sargassum) 15 g, Earthworm (Dilong, Lumbricus) 15 g, Liquoric Root (Gancao, Radix Glycyrrhizae) 15 g, Rehmannia (Dihuang, Radix Rehmanniae Glutinosae) 30 g, Chinese Gentian Root (Longdancao, Radix Gentianae Longdancao) 30 g, and Achyranthes Root (Niuxi, Achyranthis Bidentatae Radix) 30 g.
Li, 2014	LDXGD	Chinese Gentian Root (Longdancao, Radix Gentianae Longdancao) 15 g, Gardenia (Zhizi, Fructus Gardeniae Jasminoidis) 15 g, Rehmannia (Dihuang, Radix Rehmanniae Glutinosae) 10 g, Baical Skullcap Root (Huangqin, Radix Scutellariae Baicalensis) 10 g, Thorowax Root (Chaihu, Radix Bupleuri) 10 g, Plantain Seed (Cheqianzi, Semen Plantaginis) 10 g, Alisma (Zexie, Rhizoma Alismatis) 10 g, Akebia Stem (Mutong, Caulis Akebiae) 10 g, Chinese Angelica Root (Danggui, Radix Angelicae Sinensis) 5 g, and Liquoric Root (Gancao, Radix Glycyrrhizae) 5 g.
Yu and Ma, 2004	modified LDXGD	Chinese Gentian Root (Longdancao, Radix Gentianae Longdancao) 15 g, Plantain Seed (Cheqianzi, Semen Plantaginis) 30 g, Alisma (Zexie, Rhizoma Alismatis) 30 g, Rehmannia (Dihuang, Radix Rehmanniae Glutinosae) 15 g, Chinese Angelica Root (Danggui, Radix Angelicae Sinensis) 10 g, Akebia Stem (Mutong, Caulis Akebiae) 15 g, Achyranthes Root (Niuxi, Achyranthis Bidentatae Radix) 15 g, Earthworm (Dilong, Lumbricus) 10 g, Prunella (Xiakucao, Spica Prunellae Vulgaris) 30 g, and Seaweed (Haizao, Sargassum) 15 g.
He and Wang, 2010	LDXGD	Chinese Gentian Root (Longdancao, Radix Gentianae Longdancao) 9 g, Baical Skullcap Root (Huangqin, Radix Scutellariae Baicalensis) 12 g, Gardenia (Zhizi, Fructus Gardeniae Jasminoidis) 12 g, Thorowax Root (Chaihu, Radix Bupleuri) 9 g, Alisma (Zexie, Rhizoma Alismatis) 12 g, Plantain Seed (Cheqianzi, Semen Plantaginis) 9 g, Rehmannia (Dihuang, Radix Rehmanniae Glutinosae) 9 g, Akebia Stem (Mutong, Caulis Akebiae) 9 g, Chinese Angelica Root (Danggui, Radix Angelicae Sinensis) 6 g, and Liquoric Root (Gancao, Radix Glycyrrhizae) 6 g. If severe headache and vertigo were identified, Prunella (Xiakucao, Spica Prunellae Vulgaris) 9 g or Abalone Shell (Shijueming, Haliotidis Concha) 6 g was added. If constipation was identified, Rhubarb Root and Rhizome (Dahuang, Radix Et Rhizoma Rhei) 6 g was added. If deficiency of yin was identified, Ningpo Figwort Root (Xuanshen, Radix Scrophulariae Ningpoensis) 9 g was added.

*LDXGD, longdanxiegan decoction*.

### Risk of bias

The risk of bias was assessed based on evaluation criterion in the Cochrane handbook. Generally, methodological quality of the included trials was assessed to be low. All of the included trials declared randomization; however, no concrete methods used to generate the allocation sequence and allocation concealment were reported. None of the trials blinded the participants, personnel, and assessor. No detailed description on drop-out and withdrawal was reported. Because no protocol of trials could be obtained from the primary authors, the item of selective reporting could not be evaluated. The pre-trial estimation of sample size was not applied. We tried to contact the correspondence author for further information of the study by email or telephone; however, none have replied to our questions.

### Primary outcome measure

In this study, BP including SBP, DBP, and categorical BP was defined as primary outcome measure. All of the included trials reported the efficacy of LDXGD on BP. Continuous BP was reported in 3 trials (Ye, 2013; Li, 2014, 2015), while categorical BP in 6 trials (Yu and Ma, 2004; He and Wang, 2010; Cao et al., 2014; Chen et al., 2014; Xu, 2014; Ma, 2015). For categorical BP, China Food and Drug Administration recommended the evaluation criteria as below: “significant improvement” (DBP decreased by 10 mmHg, reaching the normal range, or DBP not returning to normal but reduced by more than 20 mmHg), “improvement” (DBP decreased by less than 10 mmHg but reaching the normal range, DBP decreased by 10 to 19 mmHg but not reaching the normal range, or SBP decreased by more than 30 mmHg), and “no improvement” (not meeting the above standards). Both “significant improvement” and “improvement” were considered to be “effective,” and “no improvement” was considered to be “ineffective.”

**Figure 2:**
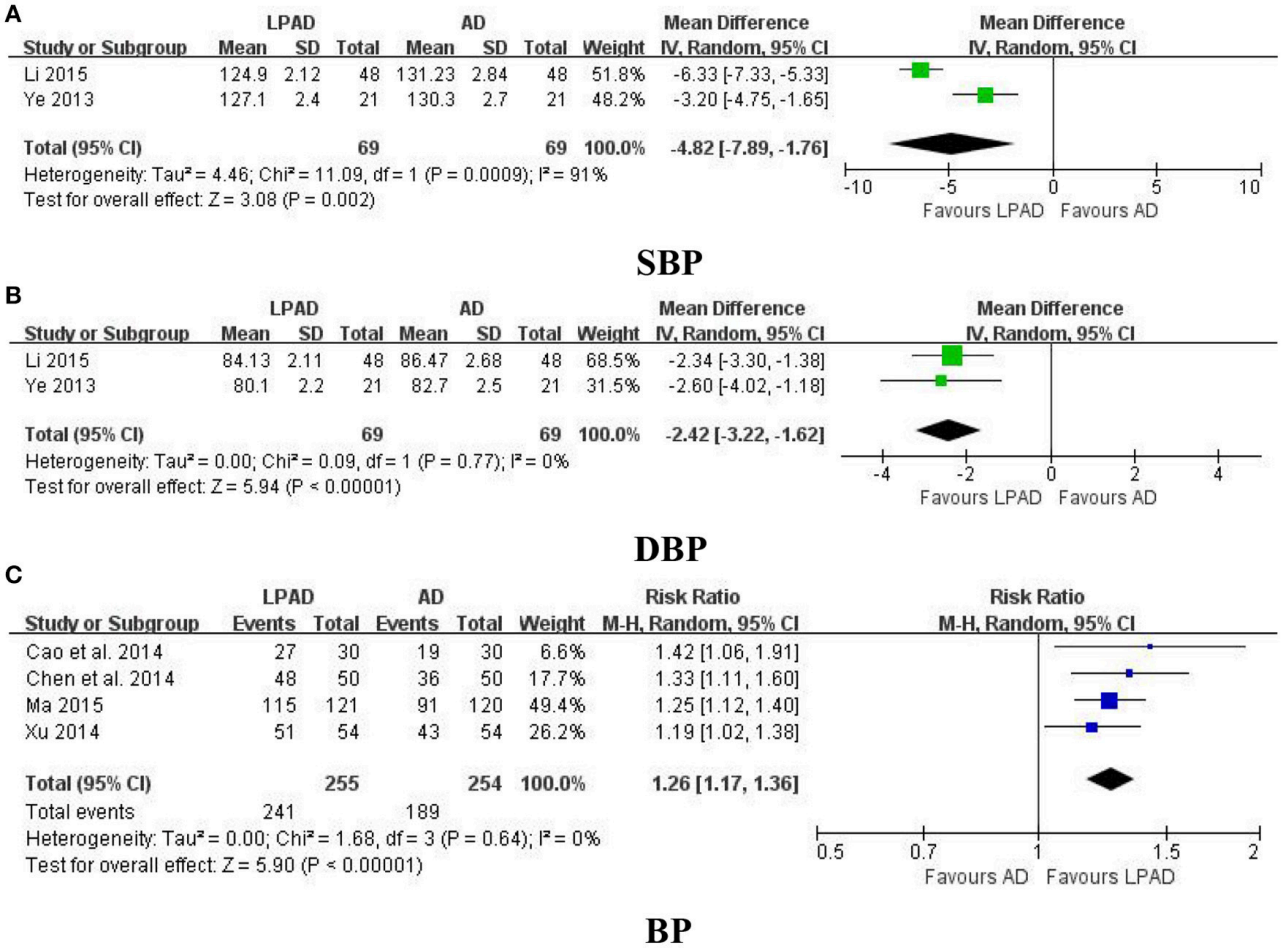
Forest plots of the comparison of LPAD vs. antihypertensive drugs for the outcomes of BP: **(A)** SBP, **(B)** DBP, and **(C)** BP. AD, antihypertensive drugs; BP, blood pressure; DBP, diastolic blood pressure; LPAD, longdanxiegan decoction plus antihypertensive drugs; SBP, systolic blood pressure.

Six trials with 647 hypertensive patients that compared LPAD with antihypertensive drugs were identified in this analysis (Ye, 2013; Cao et al., 2014; Chen et al., 2014; Xu, 2014; Li, 2015; Ma, 2015). All data were pooled and significant lowering effects of LPAD on SBP (*n* = 138; MD = −4.82 mmHg; 95% CI: −7.89 to −1.76; *P* = 0.002; Figure 2A), DBP (*n* = 138; MD = −2.42 mmHg; 95% CI: −3.22 to −1.62; *P* < 0.00001; Figure 2B), and categorical BP (*n* = 509; RR: 1.26; 95% CI: 1.17 to 1.36; *P* < 0.00001; Figure 2C) were identified when compared to antihypertensive drugs.

**Figure 3:**
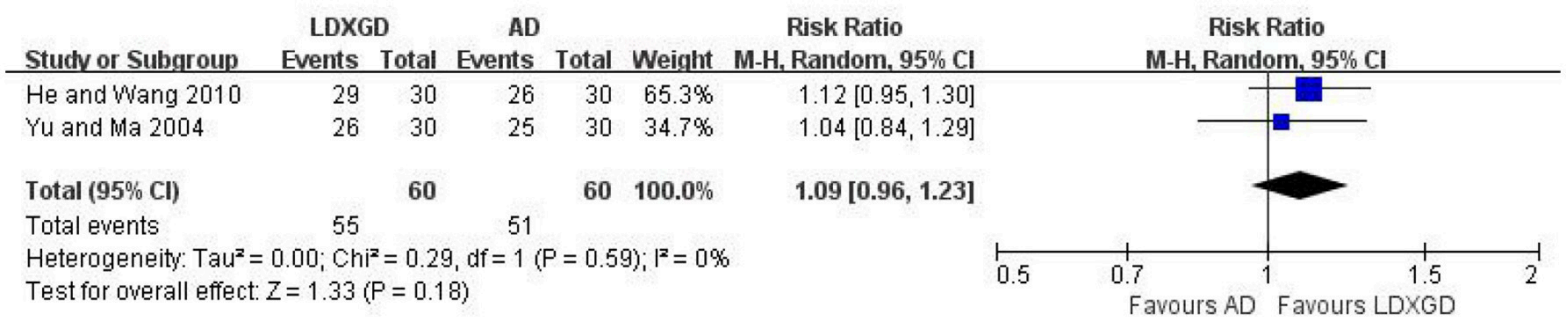
Forest plots of the comparison of LDXGD vs. antihypertensive drugs for the outcomes of BP. AD, antihypertensive drugs; BP, blood pressure; LDXGD, longdanxiegan decoction.

Three trials with 216 hypertensive patients that compared LDXGD with antihypertensive drugs were identified in this analysis (Yu and Ma, 2004; He and Wang, 2010; Li, 2014). A significant reduction favored LDXGD in SBP (*n* = 96; reduced by 5.20 mmHg) and DBP (*n* = 96; reduced by 3.60 mmHg) were identified when compared to antihypertensive drugs. But there was no difference between LDXGD and antihypertensive drugs used alone on categorical BP (*n* = 120; RR: 1.09; 95% CI: 0.96 to 1.23; *P* = 0.18; Figure 3).

### Secondary outcome measures

The secondary outcomes including mortality, progression to severe complications, symptoms, HR, TC, and TG were reported in 8 trials (Yu and Ma, 2004; He and Wang, 2010; Ye, 2013; Cao et al., 2014; Chen et al., 2014; Xu, 2014; Li, 2015; Ma, 2015).

**Figure 4:**
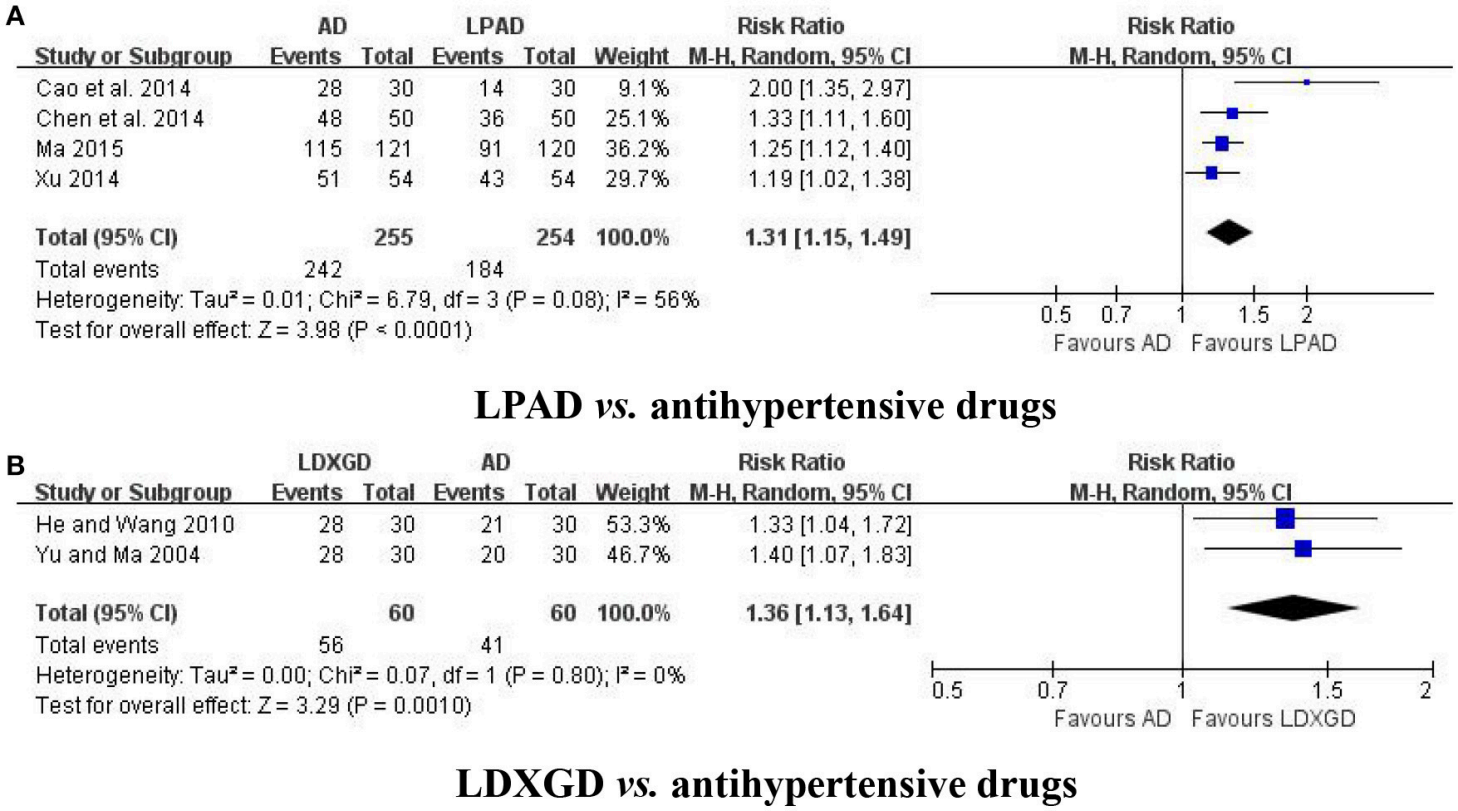
Forest plots of the efficacy of LPAD for the outcome of symptoms. AD, antihypertensive drugs; LDXGD, longdanxiegan decoction; LPAD, longdanxiegan decoction plus antihypertensive drugs. **(A)** symptoms: LPAD vs. antihypertensive drugs. **(B)** symptoms: LDXGD vs. antihypertensive drugs.

No study have reported any data on mortality or progression to severe complications. Symptoms were reported in 6 trials (Yu and Ma, 2004; He and Wang, 2010; Cao et al., 2014; Chen et al., 2014; Xu, 2014; Ma, 2015). Four trials with 509 hypertensive patients that compared LPAD with antihypertensive drugs were identified in this analysis (Cao et al., 2014; Chen et al., 2014; Xu, 2014; Ma, 2015). It was identified that improvement in symptoms was greater for LPAD than antihypertensive drugs used alone (*n* = 509; RR: 1.31; 95% CI: 1.15 to 1.49; *P* < 0.0001; Figure 4A). Two trials with 120 hypertensive patients that compared LDXGD with antihypertensive drugs were identified in this analysis (Yu and Ma, 2004; He and Wang, 2010). Meta-analysis showed that symptoms were significantly improved by LDXGD (*n* = 120; RR: 1.36; 95% CI: 1.13 to 1.64; *P* = 0.001; Figure 4B).

**Figure 5:**
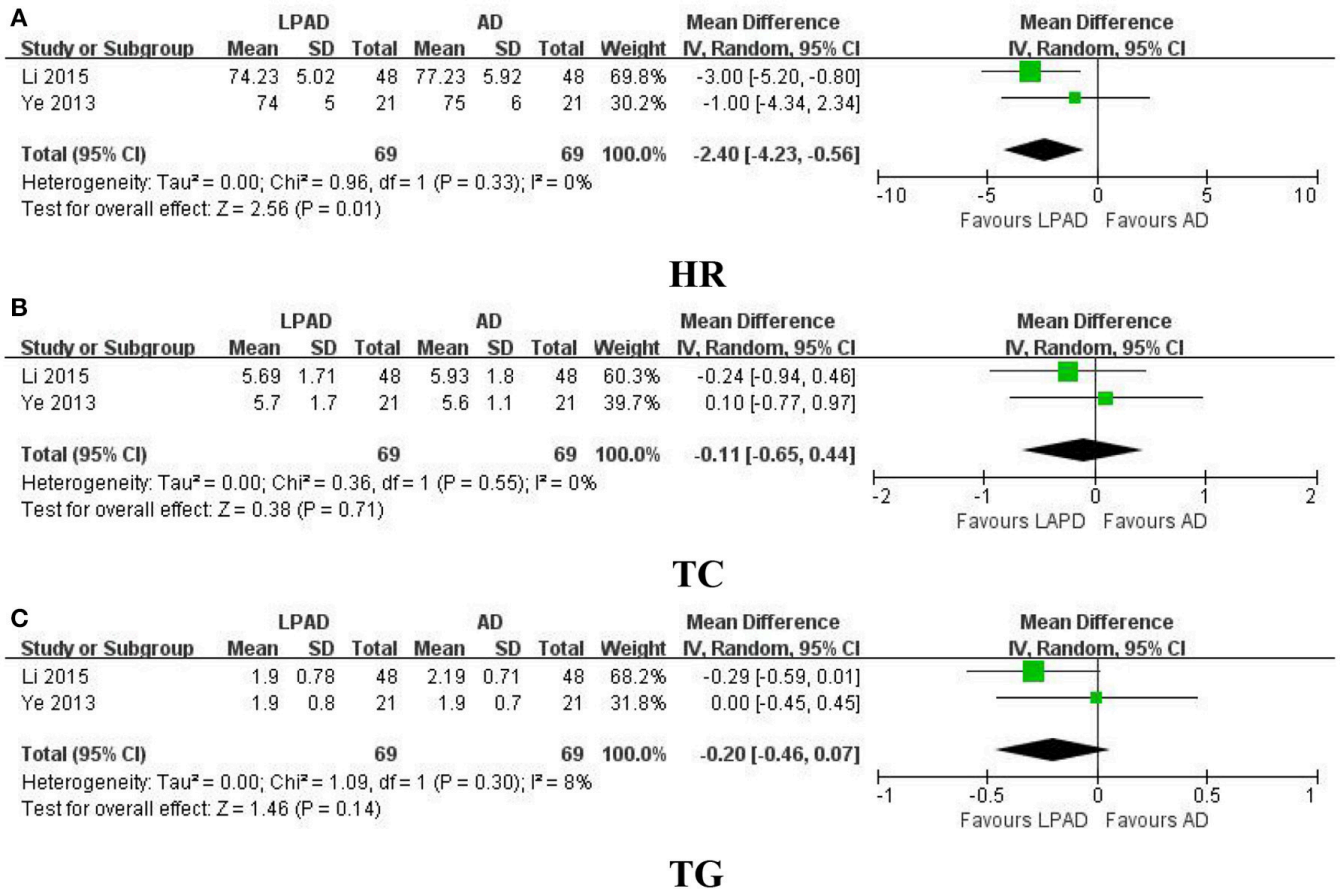
Forest plots of the comparison of LPAD vs. antihypertensive drugs for the outcomes of **(A)** HR, **(B)** TC, and **(C)** TG. AD, antihypertensive drugs; HR, heart rate; LPAD, longdanxiegan decoction plus antihypertensive drugs; TC, total cholesterol; TG, triglycerides.

HR, TC, and TG were all reported in 2 trials that compared LPAD with antihypertensive drugs (Ye, 2013; Li, 2015). It was identified that reduction in HR was greater for LPAD than antihypertensive drugs used alone (*n* = 138; MD = −2.40 bpm; 95% CI: −4.23 to −0.56; *P* = 0.01; Figure 5A). However, no significant difference between LPAD and antihypertensive drugs on TC (*n* = 138; MD = −0.11 mmol/l; 95% CI: −0.65 to 0.44; *P* = 0.71; Figure 5B) and TG (*n* = 138; MD = −0.20 mmol/l; 95% CI: −0.46 to 0.07; *P* = 0.14; Figure 5C) was identified.

### Adverse effects

**Figure 6:**
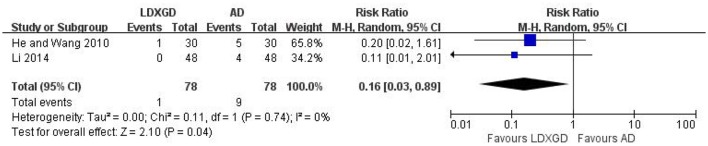
Forest plot of the reported adverse effects. AD, antihypertensive drugs; LDXGD, longdanxiegan decoction.

Adverse effects were reported in 3 trials (Yu and Ma, 2004; He and Wang, 2010; Li, 2014). No adverse effects of LDXGD was identified in 2 trials (Yu and Ma, 2004; Li, 2014). Only 1 trial reported diarrhea in the LDXGD treatment group (He and Wang, 2010). However, all of these 3 trials reported adverse effects of the antihypertensive drugs groups, including headache, facial flushing, palpitation, drowsiness, and fatigue. All of these adverse effects were not severe. No serious adverse effects were reported. Meta-analysis suggested that LDXGD was more likely to have less adverse effects than the antihypertensive drugs used alone (*n* = 156; RR: 0.16; 95% CI: 0.03 to 0.89; *P* = 0.04; Figure 6). As most of the included trials did not report any information on adverse effects, the safety of LDXGD is still unclear.

### Evaluation of publication bias

Because the number of included trials was too small (less than 10), it was impossible to conduct a sufficient additional analysis of publication bias.

## Discussion

### Summary of evidence

TCM classic herbal formulas have been recommended as complementary and alternative regimens for hypertension treatment in China and other countries (Xiong et al., 2013c, 2017b; Xiong, 2015). Due to concerns about the long term medication and adverse effects of the antihypertensive drugs, some mild to moderate hypertensive patients who are not willing to take antihypertensive drugs will give priority to herbal medicine including LDXGD either used alone or in combination with antihypertensive drugs. In their opinion, LDXGD could improve symptoms, reduce BP fluctuation, and then reduce the amount of conventional western medicine in spite of its bitter and slightly sweet taste. In addition, as it has been used for hundreds of years, LDXGD seems to be relatively safe. However, the efficacy and safety of LDXGD on hypertension is still not well-known. In this study, both LDXGD original prescription and modified LDXGD were enrolled. From the TCM physicians' viewpoint, each TCM classic herbal formula including LDXGD, is considered to be an independent “unit.” That is to say, the drug composition and efficacy of LDXGD are fixed. However, LDXGD could be modified based on the chief complaint and accompanying symptoms of the patients when seeing a doctor on the basis of syndrome differentiation and personalized treatment principle. As Chinese Gentian Root (*Longdancao*, Radix Gentianae *Longdancao*) is one of the most critical herb in LDXGD (also named “*Jun*” herb in TCM), it can't be removed when modifying the formula. However, some herbs beside Chinese Gentian Root (*Longdancao*, Radix Gentianae *Longdancao*) in LDXGD could be removed when it is no longer needed. On the other hand, other herbs could also be added into LDXGD when it is needed. With the similar composition and pharmacological effects, all of them can be considered to be the same category of LDXGD.

Our primary finding was that LDXGD either used alone or as adjuctive therapy resulted in a statistically significant reduction in BP. As we know, lowering BP is the cornerstone for the treatment of hypertension. In this review, LPAD significantly improved SBP (MD = −4.82 mmHg; 95% CI: −7.89 to −1.76; *P* = 0.002) and DBP (MD = −2.42 mmHg; 95% CI: −3.22 to −1.62; *P* < 0.00001), compared with antihypertensive drugs; when used alone, LDXGD also significantly improved SBP (reduced by 5.20 mmHg) and DBP (reduced by 3.60 mmHg), compared with antihypertensive drugs. Although the magnitude of BP reduction is small, LDXGD could be used as complementary and alternative approach for hypertensive patients, especially if they are partially insensitive to the antihypertensive drugs treatment alone and have poor compliance.

The second finding of this paper was that LDXGD significantly improve hypertension related symptoms. Vertigo and headache are common symptoms of hypertension. Although the elevated BP can be well-controlled by antihypertensive drugs, these symptoms still troubled partially hypertensive patients. Therefore, they hope to find a better therapeutic modality to improve their symptoms. In this study, LDXGD either used alone (RR: 1.36; 95% CI: 1.13 to 1.64; *P* = 0.001) or in combination with antihypertensive drugs (RR: 1.31; 95% CI: 1.15 to 1.49; *P* < 0.0001) could relieve discomfort in hypertensive patients. It suggested that LDXGD may be suitable for hypertensive patients with uncontrolled symptoms.

The third finding of this meta-analysis was the efficacy of LDXGD on HR and blood lipids. HR is an important factor in the formation process of hypertension. Lowering HR could contribute to the treatment of hypertension. Although HR was significantly reduced by LDXGD treatment (MD = −2.40 bpm; 95% CI: −4.23 to −0.56; *P* = 0.01), it should be considered with caution considering the intensity was so mild. Additionally, LDXGD as adjuctive therapy also produced a clinically meaningful, but not statistically significant, reduction in TC (MD = −0.11 mmol/l; 95% CI: −0.65 to 0.44; *P* = 0.71) and TG (MD = −0.20 mmol/l; 95% CI: −0.46 to 0.07; *P* = 0.14).

### Limitations for the review

There are still some limitations in this review that should be considered before accepting the findings. Firstly, the methodological quality of the included trials was assessed to be generally poor based on the Cochrane handbook. Essentially, no well-designed RCTs with high quality could be identified. Secondly, information recording the efficacy of LDXGD on major cardiovascular events is lacking. Therefore, whether a long-term protective effect existed is still unknown. Thirdly, the relationship between the dosage and course and BP-lowering effect of LDXGD could not be determined in this study. Fourthly, as no more detailed information on washout period and measurement method of BP by trained clinical personnel could be identified and short treatment duration to see antihypertensive effect in some trials, the efficacy of LDXGD on BP should be considered with caution. Fifthly, as RCTs published in China are more inclined to report positive results in both symptoms and serious adverse effects, the potential reporting bias can't be ignored. Sixthly, conclusions about the safety of LDXGD is still unclear as only 3 trials reported data on adverse effects.

## Conclusion

In summary, GRADE approach was used to assess the quality of LDXGD for hypertension in this study. However, the quality level of evidence was rated to be low considering the risk of bias (degraded by 2 levels), as well as potential publication bias. Therefore, due to poor methodological quality of the included trials, as well as potential reporting bias, our review found no conclusive evidence for the effectiveness of LDXGD in treating hypertension. The potential beneficial effects and safety of LDXGD should be assessed in future properly designed trials in compliance with the CONSORT Statement (Gagnier et al., 2006; Schulz et al., 2012).

## Author contributions

XX conceived the idea for this study and wrote the paper. XY, WL, LD, PW, and HY conducted the literature searching and assessed their methodological quality. Disagreement was resolved by discussion between XL and SW. All of the authors approved the final version of the article.

### Conflict of interest statement

The authors declare that the research was conducted in the absence of any commercial or financial relationships that could be construed as a potential conflict of interest.
